# Supplementary material for: Longitudinal progression of blood biomarkers reveals a key role of astrocyte reactivity in preclinical Alzheimer’s disease
Source: medRxiv. 2024 Jan 26:2024.01.25.24301779. Preprint. [Version 1] doi: 10.1101/2024.01.25.24301779 (PMC10854357; doi:10.1101/2024.01.25.24301779)
Supplement: Supplement 1 [file media-1.pdf]

Supplementary Table 1. Covariates adjusted means (standard errors) for AD converters, CU and their differences across all biomarkers at each timepoint.

	Time	Control	Case	difference<br>(Case – control)	Effect size
GFAP	10 years prior	162.00 (7.20)	197.88(7.20)	35.88 (9.04)<br>P < .0001	0.52
	5 years prior	186.21 (7.40)	217.74 (7.38)	31.53 (9.33)<br>P = 0.0008	0.34
	Onset	195.72 (8.48)	241.80 (8.52)	46.09 (11.01)<br>P < .0001	0.47
AB ratio	10 years prior	0.053 (0.0012)	0.050 (0.0012)	-0.0030(0.0016)<br>P = 0.062	-0.25
	5 years prior	0.053 (0.0011)	0.051 (0.0011)	-0.0025 (0.0014)<br>P = 0.074	-0.23
	Onset	0.052 (0.0012)	0.050 (0.0012)	-0.0018 (0.0015)<br>P = 0.21	-0.16
pTau181	10 years prior	6.39 (0.56)	7.13 (0.57)	0.74 (0.73)<br>P = 0.31	0.13
	5 years prior	8.33 (0.54)	9.07 (0.53)	0.75 (0.69)<br>P = 0.28	0.13
	Onset	9.66 (0.57)	11.29 (0.57)	1.63 (0.73)<br>P = 0.025	0.25
pTau231	10 years prior	8.95 (0.64)	9.74 (0.65)	0.80 (0.83)<br>P = 0.34	0.14
	5 years prior	12.16 (0.61)	12.73 (0.61)	0.57 (0.78)<br>P = 0.47	0.081
	Onset	13.75 (0.65)	15.39 (0.65)	1.65 (0.83)<br>P = 0.049	0.23
Nfl	10 years prior	23.56 (0.95)	25.16 (0.95)	1.61 (1.21)<br>P = 0.19	0.16
	5 years prior	28.09 (1.03)	28.20 (1.03)	0.11 (1.33)<br>P = 0.93	0.0089
	Onset	32.90 (1.24)	36.47 (1.25)	3.56 (1.65)<br>P = 0.032	0.22

Model covariates included age, sex, race and eGFR
Alzheimer’s disease (AD); Cognitively Unimpaired (CU); Glial Fibrillary Acidic Protein (GFAP); Neurofilament-Light chain (NfL); Beta-Amyloid (Aβ); Aβ-42/Aβ-40 (Aβ ratio); Phosphorylated Tau (p-tau)

Supplementary Table 2. Covariates adjusted odds ratios (per IQR change in biomarker) in predicting AD converters for each biomarker at each time point:

OR (95% CI)	10 year prior	5 year prior	Onset
GFAP	2.24 (1.37, 3.66) P = 0.0013	1.56 (1.14, 2.15) P = 0.0062	2.12 (1.50, 3.02) P < 0.0001
Ab ratio*	1.45 (1.01, 2.09) P = 0.0453	1.33 (0.951, 1.86) P = 0.0953	1.19 (0.833, 1.71) P = 0.335
Ptau181	1.18 (0.872, 1.59) P = 0.286	1.19 (0.897, 1.57) P = 0.229	1.39 (1.05, 1.85) P = 0.0215
Ptau231	1.23 (0.852, 1.77) P = 0.270	1.10 (0.827, 1.46) P = 0.516	1.44 (1.04, 2.00) P = 0.0291
Nfl	1.24 (0.768, 1.99) P = 0.385	0.938 (0.664, 1.32) P = 0.716	1.39 (1.02, 1.89) P = 0.0367

*The sign of the Aβ ratio was reversed so that its odds ratios were in the same direction as other biomarkers for ease of visualization/ interpretation.
Model covariates included age, sex, race and eGFR.

Alzheimer's disease (AD); Cognitively Unimpaired (CU); Glial Fibrillary Acidic Protein (GFAP); neurofilament-light chain (NfL); Beta-Amyloid (Aβ); Aβ-42/Aβ-40 (Aβ ratio); Phosphorylated Tau (p-tau); Odds ratio (OR); Confidence Interval (CI)

Supplementary Table 3. Partial spearman correlations among all plasma biomarkers at AD symptom onset adjusting for age and sex

Rho<br>P value	GFAP	AB ratio	Nfl	pTau181	pTau231
GFAP	1	-0.25<br><.0001	0.50<br><0.0001	0.34<br><0.0001	0.35<br><0.0001
AB ratio		1	-0.10<br>0.12	-0.16<br>0.019	-0.13<br>0.048
Nfl			1	0.36<br><0.0001	0.43<br><.0001
pTau181				1	0.74<br>< .0001
pTau231					1

Model covariates included age and sex.

Alzheimer's disease (AD); Cognitively Unimpaired (CU); Glial Fibrillary Acidic Protein (GFAP); neurofilament-light chain (NfL); Beta-Amyloid (Aβ); Aβ-42/Aβ-40 (Aβ ratio); Phosphorylated Tau (p-tau)

Supplementary Table 4. Covariates adjusted means and differences in plasma biomarkers by APOE genotype within AD converter and CU groups

		CU			AD converter		
		APOE e4-	*APOE e4+*	difference	*APOE e4-*	*APOE e4+*	difference
GFAP	-10 years	163.40 (8.23)	158.88 (14.39)	-4.51 (15.76) P = 0.775	190.38 (8.37)	213.77 (13.32)	23.40 (15.05) P = 0.121
	-5 years	184.93 (8.21)	180.79 (15.50)	-4.13 (16.79) P = 0.806	207.14 (8.53)	244.37 (13.39)	37.24 (15.23) P= 0.0149
	onset	194.71 (9.49)	204.69 (18.69)	9.98 (20.34) P = 0.624	233.33 (9.94)	266.51 (16.24)	33.19 (18.46) P = 0.0731
Ab ratio	-10 years	0.0532 (0.00136)	0.0507 (0.00251)	-0.00254 (0.00279) P = 0.363	0.0513 (0.0014)	0.0468 (0.00221)	-0.00447 (0.00251) P = 0.075
	-5 years	0.0539 (0.00124)	0.0508 (0.00221)	-0.00306 (0.00245) P = 0.211	0.0514 (0.00126)	0.0491 (0.00194)	-0.00234 (0.00222) P = 0.293
	onset	0.0532 (0.00127)	0.0471 (0.00243)	-0.00607 (0.00266) P = 0.0228	0.0508 (0.0013)	0.0496 (0.00212)	-0.00117 (0.00238) P = 0.625
pTau181	-10 years	6.47 (0.62)	5.58 (1.07)	-0.88 (1.19) P = 0.458	6.95 (0.63)	7.50 (1.03)	0.55 (1.17) P = 0.635
	-5 years	8.42 (0.59)	7.98 (1.06)	-0.44 (1.17) P = 0.707	8.65 (0.60)	10.25 (0.97)	1.60 (1.09) P = 0.144
	onset	9.72 (0.61)	9.24 (1.16)	-0.48 (1.27) P=0.703	10.66 (0.64)	12.92 (1.04)	2.26 (1.18) P = 0.0556
pTau231	-10 years	9.32 (0.72)	8.27 (1.23)	-1.05 (1.37) P = 0.446	8.98 (0.73)	11.73 (1.20)	2.75 (1.36) P = 0.044
	5 years	12.27 (0.68)	11.69 (1.22)	-0.58 (1.35) P = 0.669	11.67 (0.69)	15.30 (1.11)	3.63 (1.26) P = 0.0043
	onset	13.80 (0.70)	13.47 (1.35)	-0.33 (1.48) P = 0.823	14.23 (0.74)	18.82 (1.21)	4.59 (1.37) P = 0.0009
Nfl	-10 years	23.03 (1.10)	24.42 (1.87)	1.39 (2.07) P = 0.505	24.74 (1.10)	25.29 (1.78)	0.54 (2.01) P = 0.787
	-5 years	28.32 (1.14)	24.45 (2.24)	-3.87 (2.43) P = 0.113	27.44 (1.19)	28.98 (1.88)	1.55 (2.15) P=0.473
	onset	33.09 (1.41)	31.93 (2.83)	-1.16 (3.09) P = 0.709	35.97 (1.48)	37.02 (2.45)	1.05 (2.80) P = 0.707

Model covariates included age, sex, race and eGFR.

Alzheimer’s disease (AD); Cognitively Unimpaired (CU); Glial Fibrillary Acidic Protein (GFAP); neurofilament-light chain (NfL); Beta-Amyloid (Aβ); Aβ-42/Aβ-40 (Aβ ratio); Phosphorylated Tau (p-tau); APOE ε4- (e4-); APOE ε4+ (e4+)

Supplementary Table 5. Biomarker associations with postmortem-confirmed clinical diagnosis and severity of AD neuropathology

Table: biomarkers differences among dx group after adjusting for covariates

	Global Chi-square (DF)	Global p value	AD vs CN P value	ASY vs CN P value	AD vs ASY P value
Ab ratio	8.38 (2)	0.015	0.0038	0.106	0.178
GFAP	34.36 (2)	<.0001	<.0001	0.0045	0.0004
Nfl	4.63 (2)	0.099	*	*	*
pTau181	10.92 (2)	0.0043	0.0021	0.56	0.012
pTau231	11.19 (2)	0.0037	0.0036	0.94	0.0045

Table: biomarkers differences among Braak group after adjusting for covariates

	Global Chi-square (DF)	Global p value	Braak 5,6 vs 1,2 P value	Braak 3,4 vs 1,2 P value	Braak 5,6 vs 3,4 P value
Ab ratio	3.22 (2)	0.20	*	*	*
GFAP	10.19 (2)	0.0061	0.0026	0.16	0.015
Nfl	0.71 (2)	0.70	*	*	*
pTau181	14.07 (2)	0.0009	0.015	0.71	0.0003
pTau231	28.77 (2)	<.0001	<.0001	0.58	<.0001

Table: biomarkers differences among CERAD group after adjusting for covariates

	Global Chi-square (DF)	Global p value	CERAD 3 vs 0,1 P value	CERAD 2 vs 0,1 P value	CERAD 3 vs 2 P value
Ab ratio	6.71 (2)	0.035	0.015	0.041	0.70
GFAP	29.19 (2)	<.0001	<.0001	0.0034	0.0059
Nfl	0.37 (2)	0.83	*	*	*
pTau181	6.52 (2)	0.038	0.011	0.25	0.16
pTau231	8.38 (2)	0.015	0.0059	0.70	0.022

* pair-wise differences were not calculated where the global p-value was not significant (p > 0.05).

Alzheimer’s disease (AD); Cognitively Unimpaired (CU); Glial Fibrillary Acidic Protein (GFAP); neurofilament-light chain (NfL); Beta-Amyloid (Aβ); Aβ-42/Aβ-40 (Aβ ratio); Phosphorylated Tau (p-tau); diagnosis (dx); Consortium to Establish a Registry for Alzheimer’s disease (CERAD); degrees of freedom (DF); diagnosis (dx)

Supplementary Figure 1. Box plots of plasma biomarker differences by postmortem-confirmed clinical diagnosis and severity of AD neuropathology

Boxplot of biomarkers with DX after adjusting for age, sex and time to death

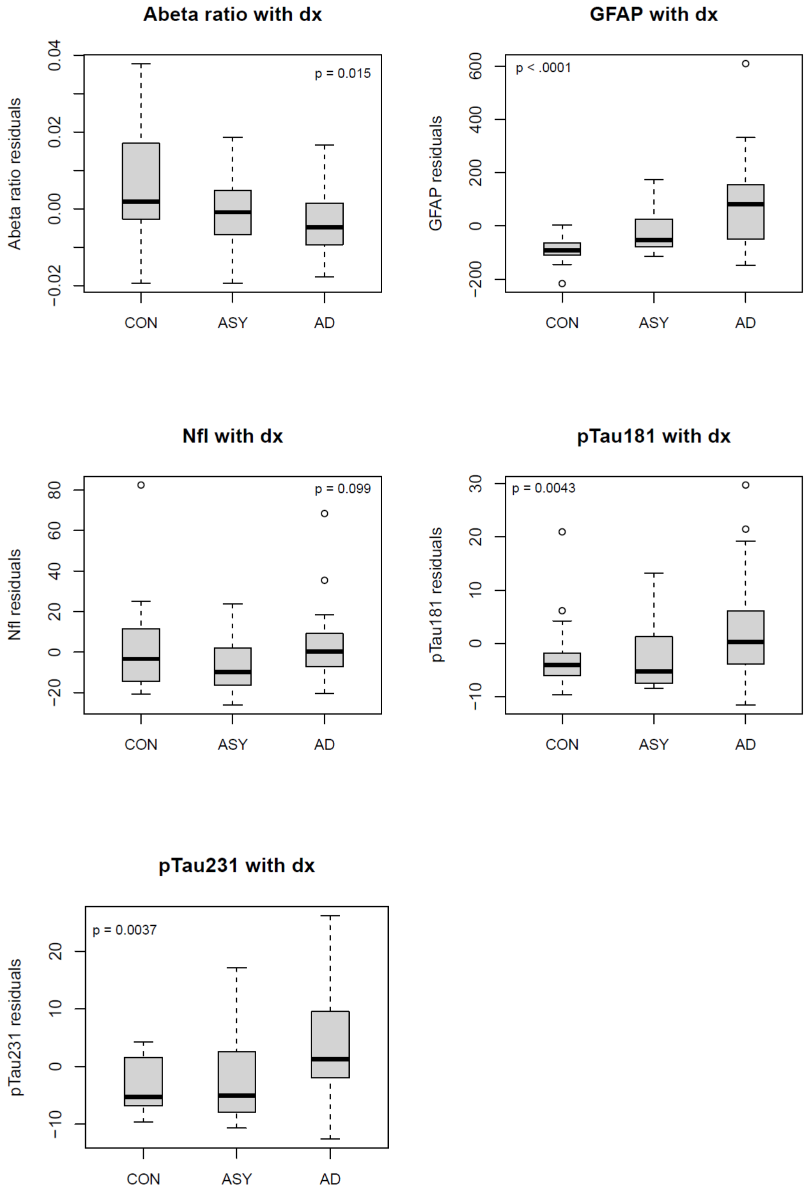

Boxplot of biomarkers with Braak scores after adjusting for age, sex and time to death

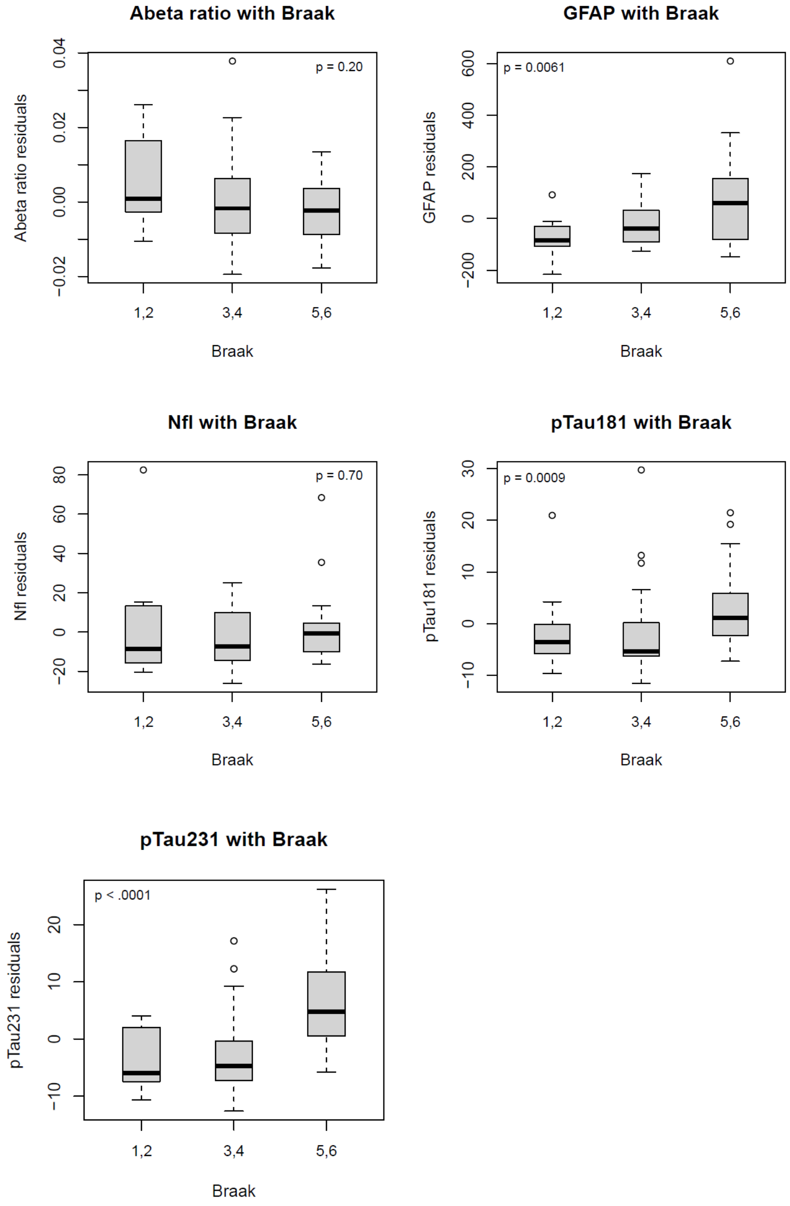

Boxplot of biomarkers with CERAD scores after adjusting for age, sex and time to death

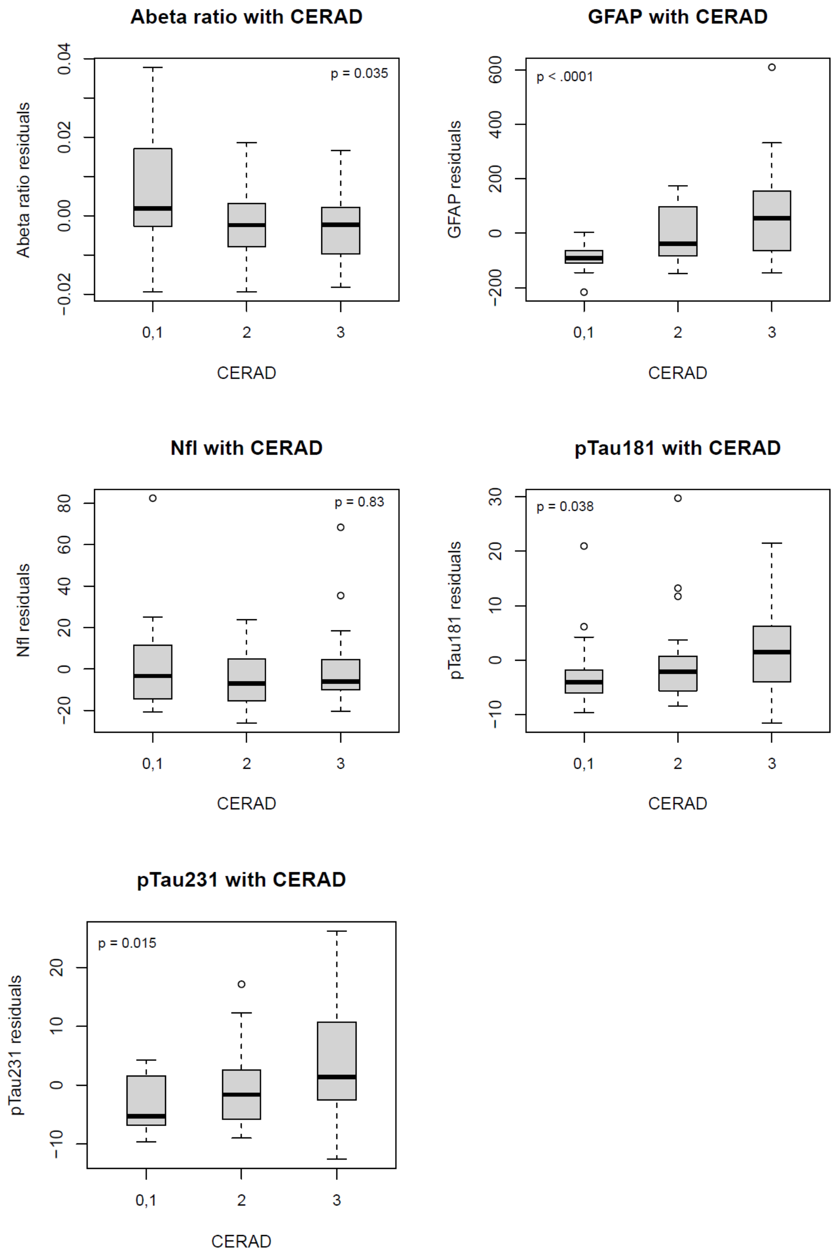

Global p-values (top left or right corner); Diagnosis (dx); Consortium to Establish a Registry for Alzheimer's disease (CERAD); Control (CON); asymptomatic Alzheimer's disease (ASY); Alzheimer's Disease (AD); Glial Fibrillary Acidic Protein (GFAP); neurofilament-light chain (NfL); Beta-Amyloid (Aβ); Aβ-42/Aβ-40 (Aβ ratio); Phosphorylated Tau (p-tau)

Supplementary Table 6. Partial spearman correlations between biomarkers and Braak, CERAD scores adjusting for age, sex and time to death:

	Spearman partial correlation	
r (P value)	Braak	CERAD
Abeta ratio	-0.27<br>p = 0.050	-0.32<br>p = 0.016
GFAP	0.43<br>p = 0.0004	0.60<br>p < .0001
Nfl	0.088<br>p = 0.49	0.063<br>p = 0.63
pTau181	0.43<br>p = 0.0004	0.31<br>p = 0.014
pTau231	0.58<br>p < .0001	0.37<br>p = 0.0034

Model covariates included age, sex, race and eGFR.

Consortium to Establish a Registry for Alzheimer's disease (CERAD); Glial Fibrillary Acidic Protein (GFAP); neurofilament-light chain (NfL); Beta-Amyloid (Aβ); Aβ-42/Aβ-40 (Aβ ratio); Phosphorylated Tau (p-tau)

Supplementary Table 7. GFAP differences between 5xFAD and WT mice

Cortex (Brain GFAP levels)

	TG - mean (SE)	WT - mean (SE)	difference (p-value)
3 mo	5.125 (1.813)	5.238 (0.992)	0.957
7 mo	57.738 (16.586)	14.313 (6.137)	0.028

	TG - mean (SE)	WT - mean (SE)	difference (p-value)
3 mo	184.15 (20.141	48.788 (3.325)	p<0.001
7 mo	673.7 (74.849)	68.513 (6.394)	p<0.001

Plasma GFAP

	TG - mean (SE)	WT - mean (SE)	difference (p-value)
3 mo	25367.81 (2988.208)	25262.5 (2817.756)	0.9797
7 mo	23230.38 (2738.476)	18748.38 (2416.069)	0.24

GFAP plasma - GFAP brain cortex

	TG - corr (p-value)	WT - corr (p-value)	Combined - corr (p-value)
3 mo	-0.3652 (0.3737)	0.1988 (0.637)	-0.1453 (0.5914)
7 mo	-0.7457 (0.0337)	-0.1634 (0.699)	-0.0916 (0.7359)

GFAP plasma - GFAP brain hippocampus

	TG - corr (p-value)	WT - corr (p-value)	Combined - corr (p-value)
3 mo	-0.136 (0.7482)	0.4607 (0.2507)	-0.1381 (0.61)
7 mo	-0.3912 (0.338)	0.5163 (0.1902)	0.3022 (0.2552)

Glial Fibrillary Acidic Protein (GFAP); months (mo); correlation (corr); standard error (SE)
